# Respiratory coinfections in COVID-19 patients evaluated by BioFire Pneumonia Panel

**DOI:** 10.1186/s43168-021-00077-8

**Published:** 2021-06-24

**Authors:** Hebatallah Hany Assal, Maged Salah, Ayman Kamal Ibrahim, Mostafa Alfishawy, Rawia Khater, Hossam Hosny Masoud, Ahmed Monier Eldemerdash, Mohamed Ali AbdelHalim

**Affiliations:** 1 Department of Chest Medicine, Faculty of Medicine, Cairo University, Al Kasr Al Aini, Old Cairo, Cairo, 11956 Egypt; 2 Department of Anaethesia, Faculty of Medicine, Cairo University, Cairo, Egypt; 3 Laboratory Department, Misr International Hospital, Cairo, Egypt; 4 Infectious Diseases Consultants and Academic Researchers of Egypt (IDCARE), Cairo, Egypt; 5 Internal Medicine department, Faculty of Medicine, Cairo University, Cairo, Egypt; 6 Intensive Care Department, Faculty of Medicine, Ain Shams University, Cairo, Egypt

**Keywords:** COVID-19, SARS-CoV-2, *Klebsiella pneumoniae*, Acinetobacter calcoaceticus baumanni complex, BioFire FilmArray Pneumonia Panel

## Abstract

**Background:**

Routine administration of antibacterials in patients with Covid-19 has been a subject of debate, with no solid data about the true prevalence of respiratory coinfections in Covid-19 patients in different geographic areas. The aim of the current study was to identify respiratory coinfections in Covid-19 patients admitted to the hospital and to identify its genetic resistance pattern using the respiratory multiplex polymerase chain reaction (PCR).

**Results:**

The study included 40 patients, 32 males (80%) and 8 (20%) females with a mean age of 59.3 ± 12.6. Half of the patients had respiratory bacterial coinfections documented by pneumonia (PN) panel. The most common isolate was *Klebsiella pneumonia*e (10/20, 50%), followed by *Acinetobacter calcoaceticus baumanni* complex (7/20, 35%). Regarding genetic resistance, thirteen (13/20, 65%) isolates were proven extended spectrum beta lactamase (ESBL)-producing Enterobacteriaceae. Thirteen (13/20, 65%) isolates were proven carbapenemase-producing organisms testing positive for New Delhi metallo-β-lactamase (NDM), oxacillinase β-lactamases (OXA-48), and Verona Integron-Encoded Metallo-β-Lactamase (VIM) (7/20, 35%; 5/20, 25%; 1/20, 5%, respectively). The four isolated *Staphylococcus aureus* were methicillin-resistant (4/20, 20%).

**Conclusion:**

In our cohort, there was 50% rate of bacterial respiratory coinfection in patients with severe Covid-19 admitted to the ICU with high rates of carbapenemase-producing gram-negative bacteria that required escalation of antibacterials and represented a challenge to clinicians.

## Background

Health care systems worldwide have been facing a real challenge with coronavirus disease 2019 (Covid-19) caused by severe acute respiratory syndrome coronavirus 2 (SARS-COV-2) with its rapid spread all over the globe. As of December 5, 2020, more than 66 million cases of Covid-19 have been reported globally, with more than 1.5 million deaths [1].

Inflammatory markers that are usually associated with bacterial infections as C-reactive protein (CRP) have been shown to be elevated in severe Covid-19 regardless of presence of bacterial coinfection [2].

The severity of presentation of critically ill patients and lack of specific antivirals together with difficulty of clinical or radiological ruling out of secondary bacterial infections had put clinicians worldwide in a great challenge [3].

Published reports using national health care associated infections surveillance data points to a high burden of multi-drug-resistant (MDR) pathogens in Egyptian health care facilities [4]. This had led to adopting the strategy of early initiation of empirical broad-spectrum antimicrobials covering MDR gram-positive and gram-negative pathogens.

Therefore, rapid pathogen detection and susceptibility profile determination are crucial. As timely action with tailored appropriate antimicrobial therapy is of extreme importance for decreasing the load on health care facilities and better clinical outcomes.

Routine diagnostic methods including culture-based pathogen detection and antimicrobial susceptibility testing have several limitations. The delayed results, in addition to the false-negative results called for the need for more rapid, sensitive, and reliable techniques for bacterial identification.

The BioFire FilmArray® Pneumonia (PN) Panel has recently been incorporated into clinical practice as a rapid and sensitive method for identifying number of typical and atypical bacterial pathogens, respiratory viruses, and several classes of antimicrobial susceptibility-associated genes directly from sputum, endotracheal aspirate (ETA), and bronchoalveolar lavage-like specimens in approximately 1 h (Table 1). The assay provides semi-quantitative results for 15 typical respiratory bacterial pathogens [5].

**Table 1:** FilmArray Pneumonia Panel plus targets

Category	Target
Typical bacteria	*Acinetobacter calcoaceticus–baumannii complex* *Enterobacter cloacae complex* *Escherichia coli* *Haemophilus influenzae* *Klebsiella aerogenes* *Klebsiella oxytoca* *Klebsiella pneumoniae group* *Moraxella catarrhalis* *Proteus* spp. *Pseudomonas aeruginosa* *Serratia marcescens* *Staphylococcus aureus* *Streptococcus agalactiae* *Streptococcus pneumoniae* *Streptococcus pyogenes*
Atypical bacteria	*Chlamydia pneumoniae* *Legionella pneumophilia* *Mycoplasma pneumoniae*
Viruses	Adenovirus Coronavirus Human metapneumovirus Human rhinovirus/enterovirus Influenza A Influenza B Middle East respiratory syndrome coronavirus (MERS-CoV) Parainfluenza virus Respiratory syncitial virus
Antimicrobial resistance genes	**Extended spectrum β-lactamase-producing Enterobacteriaceae** CTX-M **Carbapenemase-producing organisms** NDM OXA-48-like VIM IMP KPC **Methicillin-resistant** ***Staphylococcus aureus*** *mecA/C* and MREJ

The aim of the current study was to identify respiratory co infections in Covid-19 patients admitted to the hospital and to identify its genetic resistance pattern using the BioFire FilmArray method.

Furthermore, to identify the relationship of respiratory coinfections with the demographic characteristics of Covid-19 patients as well as the severity of the disease, length of hospital stay, and final outcome.

## Methods

A single-center retrospective study was done from March 2020 till October 2020. It included all adult (>18 years) patients admitted to Covid-19 ICU in Misr International Hospital (Cairo, Egypt) for acute respiratory failure related to SARS-COV-2 (RT-PCR positive for SARS-COV-2 on a nasopharyngeal swab) pneumonia. Upon admission, patients were encouraged to obtain a sputum sample for analysis. Tracheal aspirate was obtained from intubated patients. Patients that were not able to obtain a sputum sample as well as patients that had a negative RT-PCR for SARS-COV-2 on a nasopharyngeal swab were excluded from the study.

Conscious patients were given sterile cups and were encouraged to collect their respiratory secretions for microbiological sampling. Respiratory samples that were collected were sent to the microbiology laboratory where a BioFire FilmArray Pneumonia Panel was performed.

The BioFire FilmArray Pneumonia Panel (PN panel; BioFire Diagnostics, LLC) is a multiplex PCR-based diagnostic test that analyzes respiratory samples (sputum, endotracheal aspirates, bronchoalveolar lavage) and specimens for the presence of bacteria, viruses, and genetic markers of antimicrobial resistance within approximately 75 min.

It is a closed system disposable that keeps all the necessary reagents for sample preparation, reverse transcription, polymerase chain reaction (PCR), and detection in order to isolate, amplify and detect nucleic acid from multiple lower respiratory pathogens within a single respiratory specimen.

After sample collection, the user injects hydration solution and 200 μL of respiratory specimen sample combined with sample buffer into the pouch, places the pouch onto a FilmArray instrument, and starts a run. The entire run process takes about 1 h, the system also uses real-time amplification data from the assays relative to a Quantified Standard Material (QSM) included in the pouch to provide an estimated value in genomic copies per milliliter (copies/ml) for bacterial analytes.

The PN panel reports qualitative (“detected” or “not detected”) results for viral targets, bacterial targets associated with atypical pneumonia, and antibiotic resistance markers while providing a semiquantitative value for 15 additional bacterial targets commonly associated with respiratory infections (Table 1) [6].

Recently published studies investigated the agreement of semiquantitative results of the BioFire FilmArray® Pneumonia Panel test with the routine culture by comparing semiquantitative results of the PN panel (which is the number of genomic copies per milliliter) and quantitative culture (which is the colony forming unit per milliliter). The results of the studies revealed that samples that showed ≥ 10^5^ copies /ml of bacterial nucleic acids using the PN panel when cultured by conventional methods revealed significant amounts in culture.

Hence, in our study, samples showing semiquantitative results of ≥ 10^5^ genomic copies /ml were included in the study and were considered true infection [5, 6].

One hundred Covid-19 patients were admitted to our ICU. Thirty-seven of them were able to obtain a sputum sample. Bronchoscopy and bronchoalveolar lavage were done in two patients, tracheal aspirate was obtained in one patient. Blood investigations as well as results of PN panel were retrospectively reviewed.

Data collected from patients’ records were age, gender, length of hospital stay, comorbidities, mechanical ventilation, days on mechanical ventilation, type of respiratory sample, results of BioFire test, total leukocytic count, D-dimer, ferritin, C-reactive protein, absolute lymphocytic count, medication sheet, and outcome of patients.

All our patients were treated with an empiric antibiotic therapy covering methicillin-resistant *Staphylococcus aureus* (MRSA) and extended spectrum beta lactamase (ESBL) producing Gram-negative bacteria. Escalation was performed when indicated by adding colistin and increasing dose of meropenem (extended release) if a carbapenem resistant pathogen was encountered.

All patients received methylprednisolone 1–2 mg/kg/day as well as prophylactic anticoagulation. Patients with increasing inflammatory markers (ferritin, D-dimer, and CRP) and with increasing oxygen requirements with clinical signs of deterioration were given tocilizumab 8 mg/kg for maximum 2 doses 24 h apart.

The study protocol was approved by the institutional review board of Ministry of Health, Cairo, Egypt (No: 3-2021/19).

### Statistical analysis

Data was encoded and entered by using the statistical package SPSS version 21. The data were summarized by using number and percentage for qualitative variables, mean and standard deviation for normally distributed quantitative variable, and median and interquartile range for quantitative variables which are not normally distributed. Comparison between groups was done using chi-square test for qualitative variable while independent sample t-test was used for quantitative variable which are normally distributed. Non-parametrical Mann-Whitney test was used for quantitative variables which are not normally distributed. P value less than or equal to 0.05 were considered as statistically significant.

## Results

The study included 40 patients, 32 (80%) males and 8 (20%) females. Their mean age was 59.3±12.6. Twenty-two cases (55%) had underlying diseases such as kidney disease, diabetes, hypertension, or heart diseases. The mean length of hospital stay was 10±6.3 days. For mechanically ventilated patients, mean days on mechanical ventilation was 5±3.9 days. Regarding their inflammatory markers, mean white blood cell count was13.6±6.8, mean highest D-dimer level was 3190.8±4165, mean highest ferritin level 2436.7±3287.4, and mean highest CRP 163.3±121.5. Twelve patients (30%) were mechanically ventilated (Table 2).

**Table 2:** Demographic data of the studied patients

Age^†^	59.3±12.6
Male to female	4:1
Main comorbidities	
Diabetes	11
Hypertension	11
Ischemic heart disease	9
Renal impairment	2
Parkinsonism	1
APACHE II score*	10 (4.5–17)
Lab data at ICU admission:	
Highest D dimer* (ng/ml)	1021 (610–5346)
Ferritin* (ng/ml)	1366 (725–2528)
CRP* (mg/l)	133.9 (54.3–262.3)
Lymphocytes* (absolute value/μL)	506 (326–1052)
TLC^†^ (x10^3^/μL)	13.6±6.8
ALT* U/L	63.5 (37.8–129.8)
AST* U/L	66 (39–107.5)
Total Bilirubin*(mg/dl)	0.6 (0.4–0.8)
Platelets^†^ (x10^3^/μL)	342.8±110.6
Urea* mg/dl	66(50-141)
Creatinine* mg/dl	1.1 (0.9–1.3)
Procalcitonin* ng/dl	0.1 (0.1–0.5)
Outcomes in ICU	
Mechanical ventilation	12/40
ICU mortality	13/40
LOS^†^	10±6
Type of sample	
Sputum	37
BAL	2
Tracheal aspirate	1

*CRP* C-reactive protein, *TLC* total leukocytic count, *LOS* length of hospital stay, *BAL* bronchoalveolar lavage. *Data are represented by median and interquartile range. ^†^Data are represented by mean and standard deviation

Thirteen patients (32.5%) died; twenty-seven (67.5%) improved and discharged. Half of the patients had respiratory coinfections documented by PN panel. All the documented coinfections were of bacterial origin. No viruses or atypical mycobacteria were detected.

Among the 20 coinfected patients, a total of 33 bacteria were identified. The most common isolate was *Klebsiella pneumoniae* (10/20, 50%), followed by *Acinetobacter calcoaceticus baumanni complex* (7/20, 35%), *Enterobacter cloacae complex* (5/20, 25%), and *Staphylococcus aureus* (4/20, 20%); the four isolates were methicillin resistant as detected by the *mecA/C* and MREJ genes, *Streptococcus agalactia* (3/20, 15%), *Haemophilus influenza* (1/20, 5%), *Klebsiella aerogenes* (1/20, 5%), *Escherichia coli* (1/20, 5%), and *Streptococcus pneumoniae* (1/20, 5%) (Table 3).

**Table 3:** Detailed PN panel results of included patients

Patient no.	Type of sample	FA-PNEU results	Genetic resistance	Outcome
1	BAL	*Klebsiella pneumoniae ≥10*^*7*^	CTX-M, NDM, OXA-48	Death
2	BAL	-	-	Death
3	Sputum	*Klebsiella pneumoniae ≥10*^*7*^*, Acinetobacter calcoaceticus-baumanni complex10*^*5*^	CTX-M, NDM, OXA-48	Death
4	Tracheal aspirate	*Acinetobacter calcoaceticus-baumanni complex≥10*^*7*^	CTX-M, NDM, OXA-48	Death
5	Sputum	-	-	Death
6	Sputum	*Staphylococcus aureus10*^*6*^*, Enterobacter cloacae complex 10*^*5*^*, Klebsiella pneumoniae 10*^*5*^	CTX-M, OXA-48, mecA/C and MREJ	Discharge
7	Sputum	*Acinetobacter calcoaceticus-baumanni complex≥107*	NDM	Discharge
8	Sputum	-	-	Discharge
9	Sputum	-	-	Discharge
10	Sputum	-	-	Discharge
11	Sputum	*Klebsiella pneumoniae ≥10*^*7*^*, Escherichia coli ≥10*^*7*^*,*	CTX-M	Discharge
12	Sputum	*Acinetobacter calcoaceticus-baumanni complex ≥10*^*7*^*,*	NDM	Death
13	Sputum	*-*	-	Discharge
14	Sputum	*Streptococcus pneumoniae 10*^*6*^*, Streptococcus agalactiae10*^*6*^*,*	CTX-M	Discharge
15	Sputum	*Acinetobacter calcoaceticus-baumanni complex 10*^*5*^*,*	-	Discharge
16	Sputum	*Streptococcus agalactiae 10*^*6*^*, Enterobacter cloacae complex10*^*5*^*,*	CTX-M, OXA-48	Discharge
17	Sputum	-	-	Discharged
18	Sputum	-	-	Discharged
19	Sputum	*Staphylococcus aureus 10*^*5*^*, Klebsiella pneumoniae 10*^*5*^*,*	CTX-M, mecA/C and MREJ	Discharged
20	Sputum	*Staphylococcus aureus 10*^*5*^*, Klebsiella pneumoniae ≥10*^*7*^*,* *Acinetobacter calcoaceticus-baumanni complex ≥10*^*7*^*,*	CTX-M, mecA/C and MREJ	Death
21	Sputum	*-*	-	Death
22	Sputum	*-*	-	Discharge
23	Sputum	*Streptococcus agalactiae 10*^*5*^*, Klebsiella pneumoniae ≥10*^*7*^	CTX-M	Discharge
24	Sputum	*-*	-	Discharge
25	Sputum	*Staphylococcus aureus 10*^*5*^*, Enterobacter cloacae complex 10*^*5*^	mecA/C and MREJ	Discharge
26	Sputum	*Enterobacter cloacae complex 10*^*5*^*, Klebsiella pneumoniae 10*^*5*^	CTX-M,NDM,VIM	Discharge
27	Sputum	*-*	-	Discharge
28	Sputum	*-*	-	Death
29	Sputum	*Klebsiella aerogenes 10*^*6*^*, Klebsiella pneumoniae 10*^*5*^	-	Discharge
30	Sputum	*-*	-	Discharge
31	Sputum	*Acinetobacter calcoaceticus-baumanni complex≥10*^*7*^	NDM,OXA-48	Death
32	Sputum	*-*	-	Discharge
33	Sputum	*-*	-	Discharge
34	Sputum	*Enterobacter cloacae complex10*^*6*^	CTX-M	Death
35	Sputum	*-*	-	Discharge
36	Sputum	*haemophilus influenzae 10*^*6*^	-	Discharge
37	Sputum	*-*	-	
38	Sputum	*-*	-	Discharge
39	Sputum	*-*	-	Death
40	Sputum	*Klebsiella pneumoniae 10*^*6*^	CTX-M, OXA-48	Discharge

*BAL* bronchoalveolar lavage, *CTX-M* extended spectrum β-lactamase (ESBL), *NDM* New Delhi metallo-β-lactamase, *OXA-48* oxacillinase (OXA) β-lactamases, *mecA/C and MREJ* methicillin-resistant (MR) *staphylococci*, *VIM* Verona Integron-Encoded Metallo-β-Lactamase

Regarding genetic resistance, thirteen (13/20, 65%) isolates were proven ESBL-producing Enterobacteriaceae testing positive for CTX. Thirteen (13/20, 65%) isolates were proven carbapenemase-producing organisms testing positive for NDM, OXA-48, and VIM (7/20, 35%; 5/20, 25%; 1/20, 5%, consequently). The four isolated *Staphylococcus aureus* were methicillin-resistant testing positive for *mecA/C* and MREJ (4/20, 20%) (Table 3).

Considering the laboratory data, AST and TLC were found to be significantly higher in patients with documented respiratory coinfections versus patients without, p= 0.02 and 0.05 respectively. On the other hand, the other parameters showed insignificant difference between the two groups (Table 4).

**Table 4:** Comparison between patients with documented respiratory coinfection versus patients without regarding LOS and laboratory data

Variable	Documented respiratory coinfection (n=20)	No documented respiratory coinfection (n=20)	P-value
LOS^†^	10.05±6.06	9.95±6.77	0.961^‡^
TLC^†^ (x10^3^/μL)	10.71±5.14	8.07±3.26	0.05^‡^
Ferritin* (ng/ml)	992 (266–1730)	595 (334–1241)	0.194^§^
CRP* (mg/l)	56.3 (15.8–183.9)	54.3 (3.8–122.9)	0.33^§^
Lymphocytes* (absolute value/μL)	308 (92–486)	337 (288–574)	0.337^§^
Highest D-dimer* (ng/ml)	313 (690–4985)	221 (458–3487)	0.317^§^
ALT* (U/L)	59 (34–184)	68 (39–108)	0.73^§^
AST* U/L	76 (43–129)	42 (38–72)	0.02^§^
Bilirubin* (mg/dl)	0.6 (0.3–0.9)	0.5 (0.4–0.8)	0.98^§^
Platelets ^†^(x10^3^/μL)	356.8±119.27	328.5±102.38	0.43^‡^
Urea* (mg/dl)	66 (51–173)	63 (40.5–132.5)	0.41^§^
Creatinine* (mg/dl)	1.1 (0.8–1.4)	1 (0.9–1.2)	0.61^§^
Procalcitonin* (ng/ml)	0.23 (0.07–1.14)	0.05 (0.05–0.31)	0.07^§^

*LOS* length of hospital stay, *TLC* total leukocytic count, *CRP* C-reactive protein. *Data are represented by median and interquartile range. ^†^Data are represented by mean and standard deviation. ^‡^T test. ^§^Mann-Whitney test

## Discussion

As far as we know, this is the first report focusing on microbiological sampling of respiratory tract samples to detect respiratory coinfections in Covid-19 patients admitted in an ICU in Egypt.

In our study, the most common isolate was *Klebsiella pneumoniae* (10/20, 50%), followed by *Acinetobacter calcoaceticus baumanni complex* (7/20, 35%). No infection related to atypical bacteria was detected and no viral coinfection.

Numerous reports have been published documenting respiratory coinfections in Covid-19 patients with diverse results**.**

Verroken et al. had reported (13/32, 40.6%) of Covid-19 patients with documented bacterial coinfection detected by pneumonia panel. *Staphylococcus aureus*, *Haemophilus influenza*, and *Moraxella catarrhalis* were the principal bacteria identified. As similar to our results, none of the FilmArray PN Panel tests identified atypical bacteria neither other respiratory viruses [7].

In a retrospective study in Italy, screening test was done by FilmArray PN panel, bacterial coinfection was detected in 10 of 56 SARS-CoV2-infected patients (17.8%). *Staphylococcus aureus* (7/10) was the most common pathogen. Similar to the previous report, no respiratory viruses were identified [8].

Another French study revealed (26/92, 28%) of Covid-19 patients admitted to the ICU were considered as coinfected with a pathogenic bacterium upon ICU admission. The leading involved bacteria were methicillin-sensitive *Staphylococcus aureus* MSSA, *Streptococcus pneumoniae*, *Haemophilus infuenzae*, and *Enterobacteriaceae*. No coinfection with a virus nor atypical bacteria was detected [9].

Pathogens that were prevalent in our samples were obviously different from the abovementioned reports of bacterial coinfections in Covid-19 with *Klebsiella pneumoniae* (10/20, 50%) and *Acinetobacter calcoaceticus baumanni complex* (7/20, 35%) were the most common pathogens encountered and most of them were ESBL- and carbapenemase-producing organisms.

However other reports documented community acquired pathogens such as MSSA, *Streptococcus pneumoniae*, and *Haemophilus infuenzae* to be most commonly involved with no mentioned bacterial resistance genetic patterns [7–9].

The different patterns of prevalent bacteria have several explanations.

First, the samples were not all collected during the first hours of admission that could show initial disease presentation. As it was reported in previous reports that most of the patients with SARS-CoV-2 pneumonia have no respiratory secretions with only 25–30% of them having sputum production [10] so not all patients were able to collect their sputum samples in the first 24 h and the samples were collected as soon as the patient was able to expectorate his sputum. Therefore, most of the isolated bacteria was after 48 h of admission so it reflects the hospital acquired pathogens.

Second, this difference in isolated bacteria is obviously attributed to the different local microbiome. A multicenter study conducted in 3 major tertiary care Egyptian hospitals analyzed the results of lower respiratory tract samples of hospitalized patients with hospital acquired pneumonia, *Klebsiella pneumoniae* was the commonest isolated gram-negative pathogen (91/128, 71.1%) followed by *Escherichia coli* (34/128, 26.6%) [4]. Another large multicenter study was done in 91 ICUs in Egyptian hospitals. There were 2688 ICU-onset infections reported during the surveillance period. The most common pathogens reported were *Klebsiella pneumoniae* (28.7%) and *Acinetobacter* (13.7%) [11].

Several causes are attributed to the high prevalence of MDR gram-negative pathogens. Limitation in implementing stewardship programs and strict infection control measures in the hospitals as well as over prescription of antibiotics, being available over the counter in outpatient settings. All of this had led to the widespread prevalence of MDR pathogens in Egyptian hospitals. Consequently, the clinicians especially the intensivist are continuously faced with challenging clinical situations.

Although there was high prevalence of bacterial coinfection in our cohort, however none of the samples tested positive for atypical bacteria, which is in line with other reports [7–9]. This question the systematic use of antimicrobials targeting atypical bacteria in these patients. This is particularly relevant since some of these drugs have been associated with acute cardiotoxicity (QT interval prolongation when co-administered with other drugs such as hydroxychloroquine) [12].

No viral coinfection was detected in our cohort of critically ill Covid-19 patients, especially no influenza viruses. This was in agreement with the previous mentioned reports [7–9]. This finding discourages the systematic prescription of an empirical antiviral treatment with neuraminidase inhibitors in critically ill patients with a confirmed SARSCoV-2 pneumonia.

Further studies on large number of patients in multicenters are needed to be carried out to detect the true prevalence of respiratory coinfections in COVID patients. Additionally, similar studies are required to be carried out selectively on outpatient settings. This will facilitate implementation of precise management strategies and detection of the most appropriate antibiotics, if any should be used, to be prescribed on outpatient settings.

Finally concomitant samples should be sent to be tested by routine conventional cultures to be compared with BioFire results in order to clearly identify specificity and sensitivity of BioFire Pneumonia Panel test.

## Conclusion

In our cohort of studied patients, we have found 50% rate of bacterial respiratory coinfection in patients with severe SARS COV-2 admitted in the ICU. The most common detected pathogens were *Klebsiella pneumoniae* (10/20, 50%) and *Acinetobacter calcoaceticus baumanni complex* (7/20, 35%) that are resistant to the extended-spectrum antibiotics. Hence, secondary bacterial coinfection is an undeniable fact in patients with SASRS-COV-2. Systemic administration of broad-spectrum antibiotics especially antibiotics covering ESBLs being the most prevalent pathogens is recommended with prompt de-escalation as soon as possible.

## Data Availability

Data are available.

## Abbreviations

COVID-19: Coronavirus disease 2019
PCR: Polymerase chain reaction
PN panel: Pneumonia panel
ESBL: Extended spectrum beta lactamase
CTX: Extended spectrum β-lactamase (ESBL)
NDM: New Delhi metallo-β-lactamase
OXA-48: Oxacillinase β-lactamases
VIM: Verona Integron-Encoded Metallo-β-Lactamase
ICU: Intensive care unit
SARS-COV-2: Severe acute respiratory syndrome coronavirus 2
CRP: C reactive protein
MDR: Multi-drug-resistant
ETA: Endotracheal tube aspirate
RT-PCR: Reverse transcriptase polymerase chain reaction
QSM: Quantified standard material
ICU: Intensive care unit
MRSA: Methicillin-resistant *Staphylococcus aureus*
*mecA/C* and MREJ: Methicillin-resistant (MR) staphylococci
MSSA: Methicillin-sensitive *Staphylococcus aureus*

## References

[1] World Health Organization. Coronavirus disease (COVID-2019) situation reports. 2020. Available at: https://www.who.int/emergencies/diseases/novel-coronavirus-2019/situation-reports/. Accessed November 25, 2020.

[2] Wan S, Xiang Y, Fang W, Zheng Y, Li B, Hu Y et al (2020) Clinical Features and Treatment of COVID-19 Patients in Northeast Chongqing. J Med Virol 21 10.1002/jmv.25783 PMC7228368 32198776

[3] Yang X, Yu Y, Xu J, Shu H, Xia J, Liu H (2020). Clinical course and outcomes of critically ill patients with SARS-CoV-2 pneumonia in Wuhan, China: a single-centered, retrospective, observational study. Lancet Respir Med.

[4] Kotb S, Lyman M, Ismail G, Abd El Fattah M, Girgis S, Etman A (2020). Epidemiology of Carbapenem-resistant Enterobacteriaceae in Egyptian intensive care units using National Healthcare– associated Infections Surveillance Data, 2011–2017. Antimicrob Resist Infect Control.

[5] Yoo IY, Huh K, Shim HJ, Yun SA, Yoo Na Chung YN (2020). Evaluation of the BioFire FilmArray Pneumonia Panel for rapid detection of respiratory bacterial pathogens and antibiotic resistance genes in sputum and endotracheal aspirate specimens. Int J Infect Dis.

[6] Buchan B, Windham S, Balada-Llasat J, Leber A, Harrington A, Ryan Relich R et al (2020) Practical comparison of the BioFire FilmArray Pneumonia panel to routine diagnostic methods and potential impact on antimicrobial stewardship in adult hospitalized patients with lower respiratory tract infections. J Clin Microbiol 58(7) 10.1128/JCM.00135-20 PMC7315039 32350045

[7] Verroken A, Scohy A, Gerard L, Wittebole X, Collienne C, Laterre P (2020). Co-infections in COVID-19 critically ill and antibiotic management: a prospective cohort analysis. Crit Care.

[8] Calcagno A, Ghisetti V, Burdino E, Trunfio M, Allice T, Boglione L, Bonora S, Di Perri G (2020 (articles in press) Coinfection with other respiratory pathogens in COVID-19 patients. Clin Microbiol Infect. 10.1016/j.cmi.2020.08.012 10.1016/j.cmi.2020.08.012 PMC7434691 32822882

[9] Contou D, Claudinon C, Pajot O, Micaëlo M, Flandre PL, Dubert M (2020). Bacterial and viral co-infections in patients with severe SARS-CoV-2 pneumonia admitted to a French ICU. Ann Intensive Care.

[10] Guan W-J, Ni Z-Y, Hu Y, Liang W-H, Ou C-Q, He J-X, Liu L, Shan H, Lei CL, Hui DSC, du B, Li LJ, Zeng G, Yuen KY, Chen RC, Tang CL, Wang T, Chen PY, Xiang J, Li SY, Wang JL, Liang ZJ, Peng YX, Wei L, Liu Y, Hu YH, Peng P, Wang JM, Liu JY, Chen Z, Li G, Zheng ZJ, Qiu SQ, Luo J, Ye CJ, Zhu SY, Zhong NS, China Medical Treatment Expert Group for Covid-19 (2020). Clinical characteristics of coronavirus disease 2019 in China. N Engl J Med.

[11] Talaat M, El-Shokry M, El-Kholy J, Ismail G, Kotb S, Hafez S (2016). National surveillance of health care–associated infections in Egypt: developing a sustainable program in a resource-limited country. Am J Infect Control.

[12] Guo D, Cai Y, Chai D, Liang B, Bai N, Wang R (2010). The cardiotoxicity of macrolides: a systematic review. Pharmazie.

